# The conserved fertility factor SPACA4/Bouncer has divergent modes of action in vertebrate fertilization

**DOI:** 10.1073/pnas.2108777118

**Published:** 2021-09-23

**Authors:** Yoshitaka Fujihara, Sarah Herberg, Andreas Blaha, Karin Panser, Kiyonori Kobayashi, Tamara Larasati, Maria Novatchkova, Hans-Christian Theussl, Olga Olszanska, Masahito Ikawa, Andrea Pauli

**Affiliations:** ^a^Research Institute for Microbial Diseases, Osaka University, Suita 565-0871, Japan;; ^b^Department of Bioscience and Genetics, National Cerebral and Cardiovascular Center, Suita 564-8565, Japan;; ^c^Research Institute of Molecular Pathology, Vienna BioCenter, Campus-Vienna-Biocenter 1, 1030 Vienna, Austria;; ^d^Vienna BioCenter PhD Program, Doctoral School of the University of Vienna and Medical University of Vienna, 1030 Vienna, Austria;; ^e^The Institute of Medical Science, The University of Tokyo, Tokyo 108-8639, Japan

**Keywords:** fertilization, sperm–egg interaction, zona pellucida, mouse

## Abstract

Fertilization is the fundamental process that initiates the development of a new individual in all sexually reproducing species. Despite its importance, our understanding of the molecular players that govern mammalian sperm–egg interaction is incomplete, partly because many of the essential factors found in nonmammalian species do not have obvious mammalian homologs. We have recently identified the lymphocyte antigen-6 (Ly6)/urokinase-type plasminogen activator receptor (uPAR) protein Bouncer as an essential fertilization factor in zebrafish [S. Herberg, K. R. Gert, A. Schleiffer, A. Pauli, *Science* 361, 1029–1033 (2018)]. Here, we show that Bouncer’s homolog in mammals, Sperm Acrosome Associated 4 (SPACA4), is also required for efficient fertilization in mice. In contrast to fish, in which Bouncer is expressed specifically in the egg, SPACA4 is expressed exclusively in the sperm. Male knockout mice are severely subfertile, and sperm lacking SPACA4 fail to fertilize wild-type eggs in vitro. Interestingly, removal of the zona pellucida rescues the fertilization defect of *Spaca4*-deficient sperm in vitro, indicating that SPACA4 is not required for the interaction of sperm and the oolemma but rather of sperm and the zona pellucida. Our work identifies SPACA4 as an important sperm protein necessary for zona pellucida penetration during mammalian fertilization.

## Significance

We show that Bouncer’s homolog in mammals, SPACA4, is required for efficient fertilization in mice. In contrast to fish, in which Bouncer is required for female fertility, SPACA4 is expressed exclusively in the sperm and is required for male fertility. SPACA4 and Bouncer present an intriguing example of homologous proteins that both play key roles in reproduction yet diverged in terms of gene expression pattern and mode of action. Overall, our work identifies SPACA4 as an important sperm protein necessary for zona pellucida penetration during mammalian fertilization. Since human SPACA4 is also expressed exclusively in sperm, we anticipate that our findings in mice will have relevance to human biology.

Fertilization is the fundamental process by which two gametes, the sperm and the egg, fuse to form a single cell, which gives rise to a new organism. Despite being essential for all sexually reproducing organisms, the molecular mechanisms that mediate sperm–egg interaction remain poorly understood. One important step toward gaining mechanistic insights into fertilization is the identification of molecules that can mediate sperm–egg interaction. Several proteins that are specifically required for gamete interaction in mammals have been identified with the help of genetic mouse models (reviewed in refs. 1–3). Required proteins on the sperm are the transmembrane proteins IZUMO1 (4, 5), SPACA6 (6, 7), TMEM95 (7–9), FIMP (10), DCST1/2 (11, 12), and the secreted protein SOF1 (8). The factors required on the egg are the tetraspanin protein CD9 (13–15) as well as the glycosylphosphatidylinositol (GPI)-anchored protein JUNO (16). The only known interacting protein pair is IZUMO1 and JUNO (16–20), which is known to mediate adhesion between sperm and egg (4, 5, 16, 19). How the other known mammalian fertilization factors enable sperm–egg binding and/or fusion remains unclear and is subject to active research.

In addition to sperm or egg surface proteins, genetic analyses also revealed the importance of proteins of the mammalian egg coat, called zona pellucida (ZP), in fertilization. ZP proteins were shown to be required for the initial step of sperm binding to the ZP as well as the subsequent block to polyspermy, which is induced by the first sperm entering the egg (21–25). While IZUMO1, CD9, SPACA6, DCST1/2, and ZP proteins have homologs in nonmammalian vertebrates, phylogenetic analyses by us and others suggest that the other known essential mammalian fertilization factors JUNO, TMEM95, FIMP, and SOF1 lack clear nonmammalian homologs (*SI Appendix*, Fig. S1) (9, 26). In line with this observation, rapid protein evolution and divergence have been noted as general hallmarks of proteins involved in reproduction (27).

This evolutionary divergence limits the direct transfer of knowledge gained from studies in invertebrates (28–31) and plants (reviewed in ref. 32) to fertilization in vertebrates. For example, the mechanistically best-understood fertilization proteins are lysin and Verl from the marine mollusk abalone (reviewed in ref. 33). Lysin is a sperm-expressed and highly abundant secreted protein, whereas Verl is an egg-coat protein that shows structural homology to mammalian ZP2 (34). Species-specific binding of lysin to Verl causes nonenzymatic disruption of the abalone egg coat, thereby allowing conspecific sperm to fertilize the egg (34–37). However, lysin has no known homolog in vertebrates, leaving it open whether a similar mechanism might contribute to mammalian sperm passage through the ZP.

Apart from the lack of clear homologs, identification of further factors required for sperm–egg interaction in vertebrates has also been hampered by the almost exclusive focus on mammalian fertilization. Mammalian fertilization occurs internally, which poses additional experimental challenges due to the low number of eggs and inaccessibility of gametes. Moreover, possible functional redundancies among fertilization factors have limited the informative value of single-gene knockout studies in mice. Accordingly, many genes that had been implicated as potential fertilization factors based on in vitro studies were later shown to be dispensable for fertilization in vivo (38–40). Other gamete-specific proteins that might play a role in fertilization have not yet been analyzed for their function in vivo, leaving their roles during mammalian fertilization unclear.

One of these gamete-specific proteins is SPACA4 (Sperm Acrosome Associated 4; also called SAMP14 [Sperm Acrosomal Membrane Associated 14]), which was initially identified by mass spectrometry in a screen for membrane-bound human sperm proteins (41). SPACA4 is particularly interesting for three reasons: First, its fish homolog Bouncer was recently shown to be essential for sperm–egg membrane interaction in zebrafish (42). Secondly, while Bouncer is expressed exclusively in the egg in fish and frogs, its closest mammalian homolog SPACA4 is expressed exclusively in the testis (41, 42). Thirdly, the incubation of human sperm with SPACA4-specific antibodies was shown to decrease the binding and fusion of sperm with ZP-free hamster eggs in vitro (41). Taken together, these observations point toward an important function of SPACA4 for mammalian fertilization.

Here, we investigate the functional relevance of SPACA4 in mammals by analyzing the phenotypic consequence of genetic loss of SPACA4 in mice.

## Results

### Murine SPACA4 Is Expressed in the Testis and Localizes to the Inner Sperm Membrane.

**Fig. 1.**
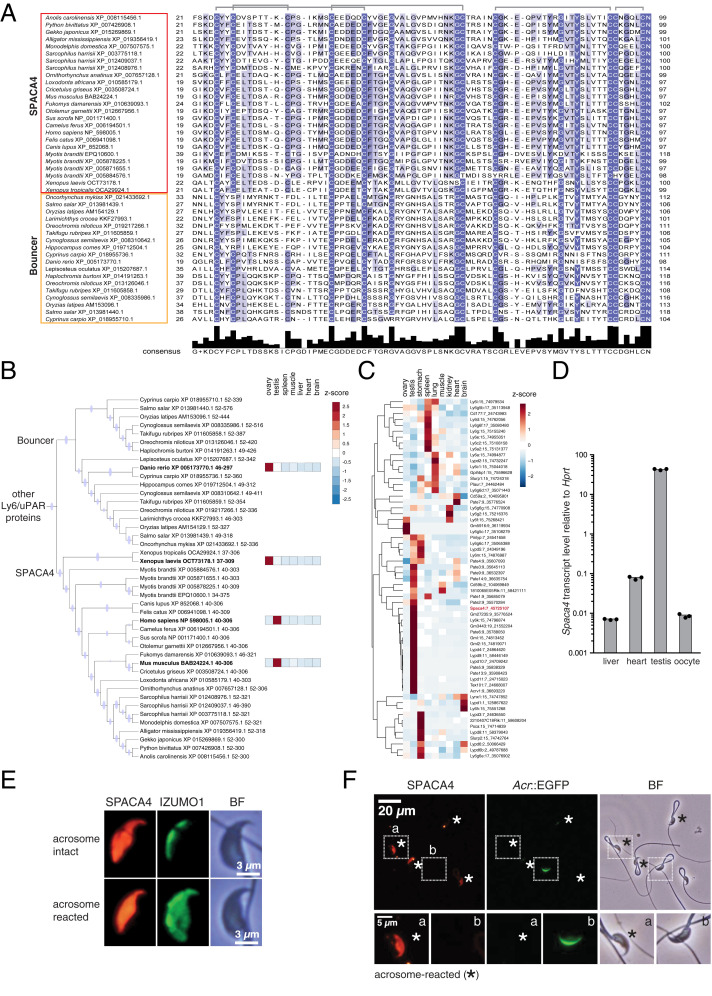
SPACA4 is expressed in murine sperm. (*A*) Protein sequence alignment of the mature domain of SPACA4/Bouncer protein family members. Shown is the mature three-finger domain of SPACA4 proteins (red box) and Bouncer proteins (orange box) lacking the N-terminal signal peptide and the carboxyl-terminal GPI-anchoring sequence. Amino acid divergence among different species is indicated based on the percent amino acid identity (blue shading); predicted cysteine bridges (gray) are shown above the alignment. Adapted from ref. 42. (*B*) Mammalian SPACA4 is the homolog for fish Bouncer and is expressed specifically in testis. Part of a maximum-likelihood phylogenetic tree based on Ly6/uPAR protein sequence alignments across vertebrates showing SPACA4 and Bouncer (refer to *SI Appendix*, Fig. S2*A* for the full tree; adapted from ref. 42). Branches supported by ultrafast bootstrap values (≥95%) are marked with a blue dot. Z-scores of averaged gene expression values across adult tissues are shown on the right for *Danio rerio* (11), *Xenopus laevis* (75), *Mus musculus* (73), and *Homo sapiens* (www.gtexportal.org). While *Bouncer/Spaca4* mRNAs are expressed in oocytes in fish and frogs, mammalian *Spaca4* mRNAs are expressed in testis. (*C*) Mouse Ly6/uPAR genes show diverse expression patterns across adult tissues. The heatmap is color coded based on z-scores of the normalized gene expression values (average of the square root) of RNA-Seq data from murine adult tissues (73). The clustering and dendrogram (*Left*) are based on expression scores. *Spaca4* is highlighted in red. Numbers behind gene names indicate chromosome location in mice. (*D*) Mouse *Spaca4* mRNA is expressed in the male germline. RT-qPCR from murine cDNA from different adult tissues reveals enrichment of *Spaca4* specifically in mouse testis. Primers amplifying *Hypoxanthineguanine phosphoribosyl transferase* (*Hprt*) were used as a control. (*E*) Mouse SPACA4 protein localizes to the sperm head. Immunostaining of SPACA4 (red) and IZUMO1 (green) under permeabilizing conditions detects SPACA4 and IZUMO1 in the sperm head of wild-type B6D2F1 mice. In contrast to IZUMO1 that relocalizes after the acrosome reaction, SPACA4 does not change its localization. BF, brightfield image. (Scale bar, 3 µm.) (*F*) Murine SPACA4 localizes to the inner sperm membrane. Immunofluorescence staining of SPACA4 (red) in mouse spermatozoa under nonpermeabilizing conditions. Spermatozoa were derived from transgenic mice expressing EGFP under the control of the *Acrosin* promoter (65), labeling sperm with intact acrosomes (green). AR spermatozoa are highlighted by an asterisk (loss of green label). Boxed areas are shown below at higher magnification (*a*: AR sperm; *b*: spermatozoa with intact acrosome). SPACA4 is detected in AR spermatozoa (*a*) but not in spermatozoa with intact acrosomes (*b*). [Scale bar, 20 µm (*Top*) and 5 µm (*Bottom*).]

Bouncer was recently discovered as an essential fertilization factor in zebrafish that is attached to the egg surface via a GPI anchor and enables sperm binding to the egg membrane (42). Evolutionary analysis revealed that Bouncer has a mammalian homolog, SPACA4 (Fig. 1 *A* and *B* and *SI Appendix*, Fig. S2*A*) (42), which raised the immediate question whether SPACA4 might also be important for mammalian reproduction. Bouncer and SPACA4 belong to the large lymphocyte antigen-6 (Ly6)/urokinase-type plasminogen activator receptor (uPAR) protein family, which is characterized by a conserved 60 to 80 amino acid protein domain containing 8 to 10 cysteines that adopt a characteristic three-finger fold (43). Most Ly6/uPAR-type genes occur in clusters in the mouse genome (*SI Appendix*, Fig. S2*B*), consistent with their origin by gene duplication (43), and are expressed in diverse tissues in mice and humans (Fig. 1*C* and *SI Appendix*, Fig. S2*C*). While mammalian *Spaca4* is not the only Ly6/uPAR-type gene expressed specifically in the male germline (testis) (Fig. 1*C* and *SI Appendix*, Fig. S2*C*), it stands out for having homologs in fish (*bouncer*) and amphibians (*Spaca4*) that are also germline specifically expressed yet in the opposite sex (ovary) (Fig. 1*B* and *SI Appendix*, Fig. S2*A*).

To confirm that *Spaca4* was indeed expressed in male but not female gametes in mice, we analyzed the expression level of *Spaca4* messenger RNA (mRNA) in different tissues using RT-qPCR. Murine *Spaca4* was detected specifically in the testis and was enriched 100- to 1,000-fold compared to other tissues (Fig. 1*D* and *SI Appendix*, Fig. S2*D*), which agrees with published RNA-sequencing (RNA-Seq) data from mice (Fig. 1 *B* and *C* and *SI Appendix*, Fig. S2*C*) and with the reported testis-specific expression in humans (41) (*SI Appendix*, Fig. S2*C*). Analysis of published single-cell RNA-Seq data from murine spermatogenesis revealed a peak of *Spaca4* mRNA expression in round spermatids, which resembles the expression of *Izumo1* mRNA in timing and magnitude (*SI Appendix*, Fig. S3) (44). Using anti-mouse SPACA4 antibodies, SPACA4 was found to localize to the sperm head (Fig. 1*E*) and was readily detected on nonpermeabilized acrosome-reacted (AR), but not on acrosome-intact, live sperm (Fig. 1*F*). This expression pattern is consistent with the reported acrosomal membrane localization of human SPACA4 (41).

### Male Mice Lacking SPACA4 Are Subfertile.

**Fig. 2.**
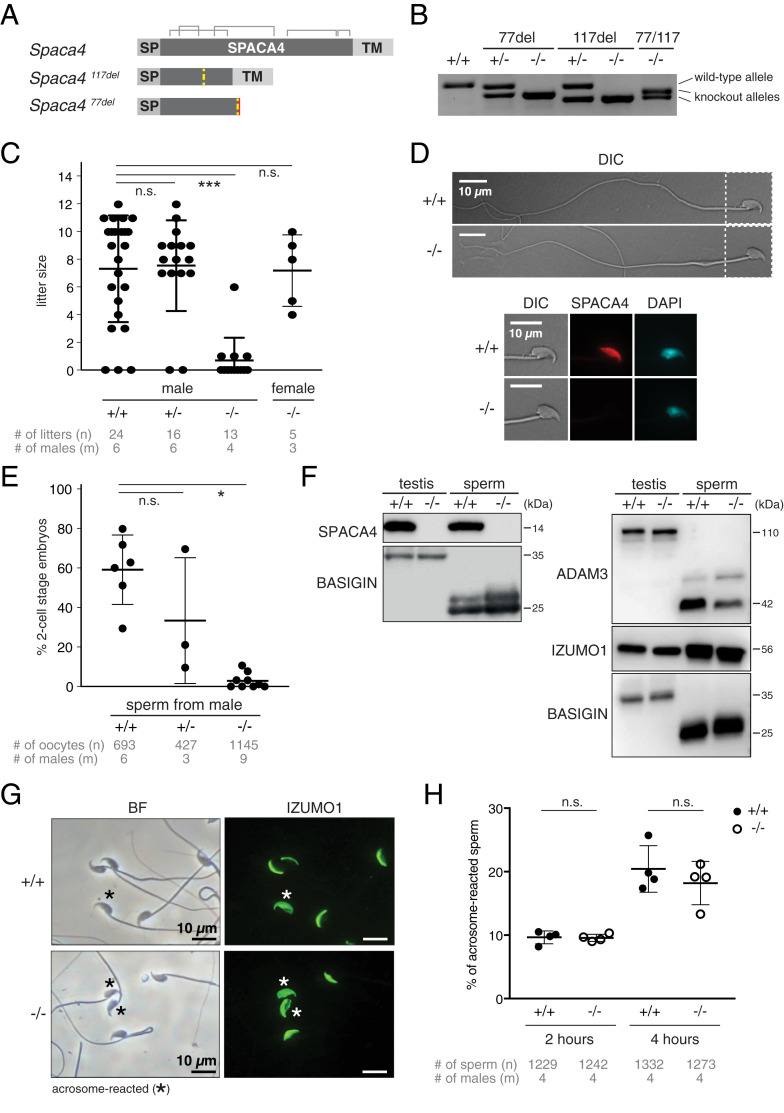
SPACA4 is required for efficient fertilization in male mice. (*A* and *B*) Overview of the C57BL/6J-*Spaca4* knockout alleles generated by CRISPR-Cas9–mediated targeted mutagenesis. One allele contains a 117-nt in-frame deletion after amino acid 42. The other allele contains a 77-nt out-of-frame deletion after amino acid 47. (*A*) Schematic of the wild-type and knockout alleles. Yellow dashed lines indicate the site of the deletions. Predicted disulfide bridges are indicated in gray. SP, signal peptide; TM, transmembrane region. (*B*) Genotyping of *Spaca4* knockout mice. *Spaca4* PCR products are separated based on their sizes on an agarose gel. (*C*) *Spaca4* knockout male mice are subfertile. Litter sizes of C57BL/6J-*Spaca4* wild-type (+/+), heterozygous (+/−), and transheterozygous (−/−) males caged with B6129F1 wild-type females or B6129F1 wild-type males caged with transheterozygous (−/−) females. Successful mating was confirmed by plug checks. Data are means ± SD. ***P < 0.0001 (Kruskal–Wallis test with Dunn multiple-comparisons test); n.s., not significant. *n* = number of litters; m = number of male mice tested. (*D*) Sperm morphology is normal in the absence of SPACA4 protein. DIC, differential interference contrast image. The sperm heads (boxed areas) are shown below at higher magnification. Immunostaining of sperm detects SPACA4 protein (red) under permeabilizing conditions in the head of sperm from wild-type (+/+) mice but not in sperm from *Spaca4* knockout (−/−) mice. DAPI (cyan) staining labels the sperm nucleus. (Scale bars, 10 µm.) (*E*) Sperm from *Spaca4* knockout mice has a severely reduced ability to fertilize wild-type oocytes. IVF of oocytes from C57BL/6J wild-type females using sperm from C57BL/6J-*Spaca4* wild-type (+/+), heterozygous (+/−), or transheterozygous (−/−) males. Plotted is the percentage of two-cell stage embryos as a measure of successful fertilization. Data are means ± SD. *P = 0.014 (Kruskal–Wallis test with Dunn multiple-comparisons test); n.s., not significant. *n* = total number of oocytes; m = number of males tested. (*F*) IZUMO1 and ADAM3 proteins are expressed and processed normally in sperm and testis from *Spaca4* knockout mice. Western blot analysis showing the absence of SPACA4 protein in testes and spermatozoa of *Spaca4* knockout mice (*Left*) and expression and processing of ADAM3 and IZUMO1 proteins similar to wild-type controls. Testes and spermatozoa of wild-type mice, as well as BASIGIN protein, which undergoes proteolytic cleavage during sperm maturation (7), are used as control. (*G*) IZUMO1 localization is normal in *Spaca4* knockout mice. Immunostaining of sperm under permeabilizing conditions shows normal localization of IZUMO1 (green) in acrosome-intact wild-type (+/+) and SPACA4-deficient (−/−) sperm and normal relocalization of IZUMO1 in AR sperm (asterisk) in both genotypes. BF, brightfield image. (Scale bars, 10 µm.) (*H*) Quantification of the percentage of AR sperm 2 or 4 h after incubation of capacitated sperm. Acrosome reaction was assessed by immunostaining for IZUMO1. Data are means ± SD; n.s., not significant. *n* = total number of sperm; m = number of males tested.

To investigate the function of mammalian SPACA4 in vivo, we generated *Spaca4* knockout mice by CRISPR-Cas9–mediated targeted mutagenesis. We recovered two mutant alleles in the C57BL/6J background. The first one, in the following called *Spaca4*^*77del*^, contains a 77-nt deletion leading to a frameshift after amino acid 42 (Fig. 2 *A* and *B* and *SI Appendix*, Fig. S4*A*). The second allele, in the following called *Spaca4*^*117del*^, contains a 117-nt in-frame deletion, which removes half (39 amino acids) of the mature SPACA4 protein, is therefore unable to fold into the characteristic three-finger conformation, and is thus also predicted to result in a full knockout mutation (Fig. 2 *A* and *B* and *SI Appendix*, Fig. S4*A*). A third, independent *Spaca4* knockout mouse (*Spaca4*^tm1Osb^) was generated in the B6D2 background by replacing the whole exon of the *Spaca4* gene with a neomycin resistance cassette using homologous recombination (*SI Appendix*, Fig. S5 *A*–*C*). *Spaca4* knockout mice appeared indistinguishable from wild-type and heterozygous littermates, revealing that SPACA4 is dispensable for somatic development in mice.

In line with SPACA4’s sperm-specific expression in mammals, we found that SPACA4 is necessary for male fertility. The litter size of transheterozygous (*Spaca4*^*117*del/*77del*^*)* as well as homozygous mutant male mice was significantly lower than the litter sizes of wild-type or heterozygous male mice (*P* < 0.0001) (Fig. 2*C* and *SI Appendix*, Figs. S4*B* and S5*D*). In contrast, female fertility was not affected by the *Spaca4* mutation (Fig. 2*C*). The observed defect in male fertility was not due to an inability of *Spaca4* knockout males to mate, as verified by the presence of vaginal plugs.

The severely reduced fertility of males lacking SPACA4 could have multiple reasons, including reduced sperm count, immotility of the sperm, or a defect in gamete interaction. Sperm morphology and numbers were similar in wild-type and knockout mice, and sperm derived from knockout males was motile (Fig. 2*D* and *SI Appendix*, Fig. S4 *C*–*E*), suggesting that SPACA4 is required during sperm–egg interaction. To test this hypothesis, we performed in vitro fertilization (IVF) experiments, which revealed that *Spaca4* mutant sperm was severely compromised in its ability to fertilize wild-type oocytes in vitro: Spermatozoa from transheterozygous (*Spaca4*^*117*del/*77del*^*)* male mice resulted in a severely reduced average fertilization rate of 2.9% (two-cell stage embryos), whereas the average fertilization rates using spermatozoa from wild-type or heterozygous (*Spaca4*^*117*del^/+ or *Spaca4*^*77del*^/+) mice were at 59.4% or 33.4%, respectively (Fig. 2*E*). Similar defects in IVF were observed in *Spaca4*^tm1Osb^ mutant sperm (*SI Appendix*, Fig. S5*E*).

The inability of SPACA4-deficient sperm to efficiently fertilize wild-type oocytes could be due to multiple reasons, including 1) a failure to passage the uterotubal junction, a defect which has been shown to correlate with the lack of the presence and/or posttranslational processing of ADAM3 (45, 46), 2) loss of expression and/or improper localization of IZUMO1, or 3) a defect in undergoing the acrosome reaction. However, Western blotting for SPACA4, ADAM3, and IZUMO1 and immunofluorescence staining for IZUMO1 revealed that ADAM3 protein was processed into the shorter protein isoform in knockout sperm (Fig. 2*F*) and that sperm of *Spaca4* knockout mice showed normal rates of acrosome reactions as well as normal expression and relocalization of IZUMO1 upon acrosome reaction (Fig. 2 *G* and *H*). Even though ADAM3 processing was slightly reduced in *Spaca4* mutant sperm compared to wild-type sperm, this decrease cannot account for the severe fertilization defect in *Spaca4* mutants given that much less of processed ADAM3 is required to rescue infertility mutants (47). Overall, we conclude that SPACA4 is an important, albeit not absolutely essential, protein required at the step of mammalian gamete interaction.

### SPACA4 Is Required for Efficient Penetration of the ZP.

**Fig. 3.**
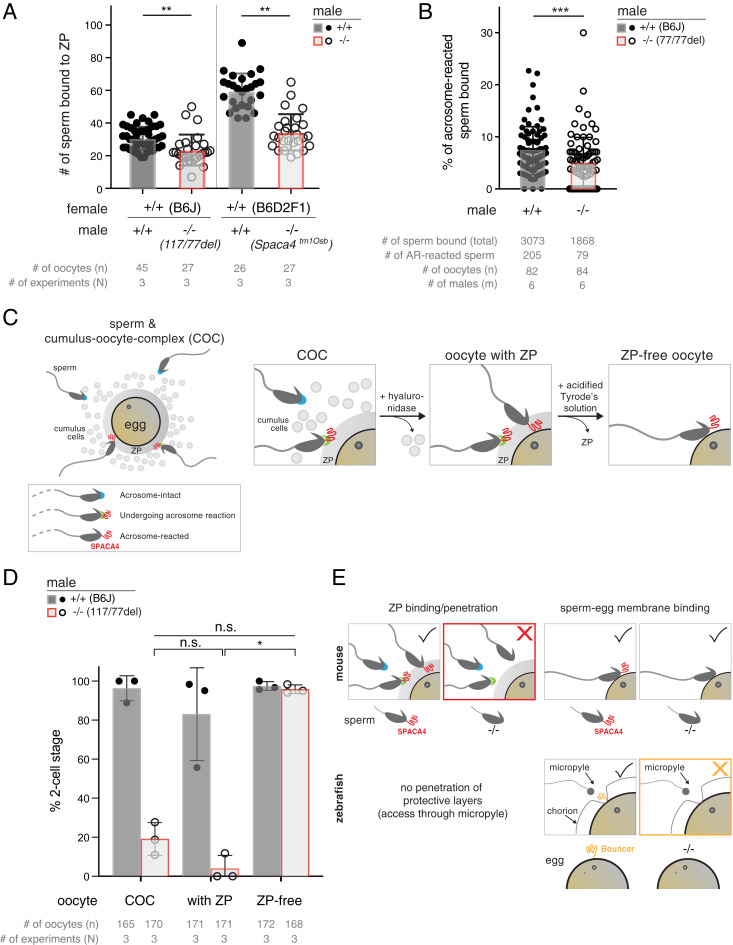
SPACA4 enables sperm to bind to and penetrate the ZP. (*A*) SPACA4 is required for efficient binding of sperm to the ZP. Sperm of the indicated genotypes was incubated for 30 min with hyaluronidase-treated oocytes (with intact ZP) of matching genetic backgrounds. Plotted is the number of sperm bound to ZP-containing oocytes. Data are means ± SD. *P* values (**P < 0.01) are by Student’s *t* test. *n* = total number of oocytes; *N* = number of replicates. (*B*) Quantification of the percentage of AR sperm derived from wild-type (+/+) and *Spaca4*^*77nt-del/77nt-del*^ (−/−) males bound to ZP-containing wild-type oocytes. Data are means ± SD. The *P* value (***P < 0.001) is by Student’s *t* test. Numbers of total sperm bound, AR sperm, oocytes (*n*) and males (m) tested are indicated (*SI Appendix*, Fig. S6 *A* and *B*). (*C*) Schematic of sperm bound to the COC and experimental treatments used to remove the cumulus cells (by treatment of COCs with hyaluronidase) and the ZP (by treatment of cumulus-free oocytes with acidified Tyrode’s solution) from the COCs. Acrosome-intact sperm, blue cap; sperm undergoing the acrosome reaction, green cap, and SPACA4 (red) getting exposed; AR sperm, no cap, and SPACA4 (red) exposed. (*D*) SPACA4 is required for ZP penetration but not for oolemma binding and fusion. IVF performed with COCs from superovulated C57BL/6J females, cumulus cell–free oocytes (oocyte with ZP), and ZP-free oocytes with sperm from either wild-type C57BL/6J males or age-matched *Spaca4*^*117nt-del/77nt-del*^ males. Plotted is the percentage of two-cell stage embryos as a measure of successful fertilization. Data are means ± SD. *P* values (*P < 0.05; n.s., not significant) are by a Kruskal–Wallis test with Dunn multiple-comparisons test. *n* = total number of oocytes; *N* = number of replicates. (*E*) Comparison of SPACA4’s function in murine fertilization versus Bouncer’s role in fish fertilization. (*Top*) In the mouse, sperm-expressed SPACA4 (red) is exposed in AR sperm (green cap and no cap) and is required for efficient ZP penetration (*Left*) but not for sperm–egg membrane binding (*Right*). Acrosome-intact sperm, blue cap. (*Bottom*) In zebrafish, sperm can access the egg membrane via the preformed funnel, the so-called micropyle, in the chorion. Egg-expressed Bouncer (orange) is required for sperm–egg membrane binding in zebrafish (42). The tick mark (yes) or cross (no) at the top right in each box indicates whether this step of fertilization can occur in the wild-type or mutant condition.

The marked IVF defect of sperm derived from *Spaca4*^−/−^ males showed that SPACA4 is necessary for sperm to efficiently interact with the egg. In mammals, this interaction occurs in two steps: Sperm first needs to bind and penetrate the ZP before binding to the egg membrane (oolemma), which enables sperm–egg fusion. To determine at which stage of fertilization SPACA4-deficient sperm is impaired, we quantified the number of spermatozoa bound to the ZP 30 min after insemination. We found that in both *Spaca4*^−/−^ mutant strains, fewer mutant spermatozoa bound the ZP of wild-type oocytes compared to control wild-type sperm (23.5 ± 9.5 in the case of *Spaca4*^*117*/*77*^ versus 30.7 ± 7.0 for wild-type [B6J] [*P* < 0.001]; 34.3 ± 11.1 in the case of *Spaca4*^tm1Osb^ versus 59.7 ± 10.7 for wild-type [B6D2F1] [*P* < 0.001]) (Fig. 3*A*). A closer analysis of the observed defect in ZP binding revealed that both acrosome-intact and AR sperm from *Spaca4* knockout males showed decreased binding to wild-type oocytes (4.5% AR sperm bound per oocyte [0.9 ± 0.8 AR sperm out of 22.2 ± 8.7 total sperm bound per oocyte] in the case of *Spaca4*^*77*/*77*^ versus 7.4% AR sperm bound per oocyte [2.5 ± 1.2 AR sperm out of 37.5 ± 11.7 total sperm bound per oocyte] in the case of wild-type [B6J] [*P* < 0.001]) (Fig. 3*B* and *SI Appendix*, Fig. S6 *A* and *B*). Analysis of sperm motility showed slightly reduced motility of mutant sperm (*SI Appendix*, Fig. S6*C*). However, given that the difference in motility between the two different wild-type strains (B6J and B6D2F1) was larger than between the mutant and background-matched wild-type spermatozoa and that sperm motility of B6D2 knockout sperm was higher than that of wild-type B6J sperm (*SI Appendix*, Fig. S6*C*), we conclude that the small decrease in motility cannot explain the defect in fertilizing ability of *Spaca4* knockout spermatozoa. Instead, the main defect of *Spaca4* mutant spermatozoa appears in sperm binding.

The reduced binding of SPACA4-deficient sperm to the ZP prompted us to ask at which step of sperm–oocyte complex interaction SPACA4 was required, since mammalian sperm first needs to penetrate the protective layers that surround the oocyte, namely, the cumulus layer and the ZP (Fig. 3*C*). To test whether removal of the cumulus cells and/or the ZP can rescue the fertility defect of SPACA4-deficient sperm in IVF, wild-type oocytes were treated with hyaluronidase and acidified Tyrode’s solution to remove the cumulus layer and ZP, respectively (48) (Fig. 3*C*). Fertilization by wild-type sperm was not significantly affected by the different treatments (Fig. 3*D*). Similarly, removal of the cumulus cells did not rescue the low fertilization rate of SPACA4-deficient sperm (18.8% cumulus–oocytle complexes [COCs] versus 4.7% [cumulus cells removed] [*P* = 0.884]; Fig. 3*D*). However, removal of the ZP enabled SPACA4-deficient sperm to fertilize zona-free oocytes at a similarly high rate (95.8%) as wild-type sperm (97.1%) (*P* value for mutant sperm fertilizing oocytes with ZP versus ZP-free = 0.033) (Fig. 3*D*). We therefore conclude that murine SPACA4 is required for the sperm’s ability to efficiently bind to and traverse the ZP (Fig. 3*E*).

## Discussion

Here, we reveal that SPACA4, the mammalian homolog of fish Bouncer (Fig. 1 *A* and *B* and *SI Appendix*, Fig. S2*A*) (42), is required for normal male fertility in mice (Fig. 2 and *SI Appendix*, Fig. S5). We find that SPACA4 is expressed in the sperm head and gets exposed by the acrosome reaction (Figs. 1 *E* and *F* and 2*D*), which enables sperm to efficiently bind to and penetrate the ZP (Fig. 3). SPACA4, therefore, differs from the proposed function of other known sperm fertility factors required for sperm–egg interaction in mice (SPACA6, IZUMO1, SOF1, FIMP, TMEM95, and DCST1/2), which are required at the later step of sperm–egg membrane binding and/or fusion (4–12). Thus, our work provides genetic evidence for an important function of SPACA4 for the step of ZP binding in mice. Together with previous SPACA4-antibody–blocking experiments with human sperm and the conserved expression pattern of SPACA4 in human sperm (41) (Fig. 1*B* and *SI Appendix*, Fig. S2 *A* and *C*), our results in mice could have direct relevance for male fertility in humans. Although both our results presented here from genetic knockout studies in mice, as well as the antibody-blocking experiments with human sperm (41), are consistent with human SPACA4 playing an important role in human reproduction, the precise function of human SPACA4 is still unclear. While our studies in mice demonstrate a role for murine SPACA4 in ZP binding, but not in the subsequent step of sperm–egg membrane interaction, the results reported by Shetty et al. for human SPACA4 suggested a role in sperm–egg membrane interaction but did not assess ZP binding (41). The difference in the reported function of SPACA4 between human and mice could be due to technical reasons [e.g., due to known difficulties in biochemical blocking studies to uncover the real physiological function of proteins, as has been observed, for example, in the case of Acrosin (49, 50), Fertilin (51, 52), and CD46 (53, 54) (reviewed in refs. 40 and 55)]. In this case, human SPACA4 may function at the step of ZP binding, as we report here for murine SPACA4. Alternatively, it could reflect a real biological difference between mouse and human SPACA4’s function. Future work will be needed to explore a possible link between *Spaca4* mutations in humans and subfertility in men presenting normal sperm count, morphology, and motility.

SPACA4 and Bouncer are not the only Ly6/uPAR proteins that have been linked to vertebrate reproduction. Several other members of this large gene family show a testis-restricted expression pattern in mammals (Fig. 1*C* and *SI Appendix*, Fig. S2*C*), some of which (*Tex101*, *Ly6k*, *Lypd4*, *Pate4*, and the *Pate* gene cluster) have been confirmed in genetic knockout studies to be required for male fertility in mice (56–58). In light of these known male-specific requirements for Ly6/uPAR proteins in mammals, SPACA4 and Bouncer present an interesting example of homologous proteins that diverged in terms of gene expression pattern and mode of action. Our phylogenetic sequence analysis shows that Bouncer and SPACA4 are the closest homologs among all other Ly6/uPAR family members (Fig. 1*B* and *SI Appendix*, Fig. S2*A*), yet they have opposing germline-specific expression patterns that broadly correlate with external (fish, amphibians; expressed in the egg) versus internal (mammals, reptiles; expressed in the sperm) fertilization (Fig. 1*B* and *SI Appendix*, Fig. S2*A*). We currently do not know how this different gene expression pattern arose or whether it evolved as a consequence of the different fertilization modes. One possibility is that an ancestral Ly6/uPAR protein might have been expressed in both male and female gonads and that sex-specific loss of expression occurred either by chance in different lineages or in response to a functional benefit of the restricted expression of SPACA4/Bouncer to the male or female germline. Acquisition of a restricted expression domain from an initially broader expression pattern has been proposed for other members of the Ly6/uPAR protein family, namely, for snake toxins that evolved a venom gland–restricted expression pattern (59).

The difference in expression pattern also extends to a difference in mode of action, at least in the case of the two example model organisms zebrafish (42) and mice (this work): While Bouncer in fish is required in the egg for sperm binding to the oolemma (42), the results presented here reveal that SPACA4 is dispensable for sperm binding to the oolemma in mice and is instead required for the preceding process of ZP penetration and/or binding. In this regard, it is interesting to note that mammalian and fish gametes differ in key aspects: Mammalian sperm has an acrosome, a specialized vesicle in the sperm head that must undergo exocytosis to expose important membrane-localized fertility factors (5, 60). This so-called acrosome reaction is important for successful ZP penetration and fertilization. Moreover, mammalian sperm needs to first bind to and penetrate the outer coat before gaining access to the egg membrane. Fish sperm, on the other hand, lacks an acrosome and has direct access to the oolemma through the micropyle, a preformed funnel in the outer protective layer of the fish egg. One can therefore speculate that acquisition of a sperm-specific expression of SPACA4 in mammals was beneficial to allow sperm to pass the additional outer barrier. In zebrafish, current data suggests that Bouncer acts in a unilateral manner by interacting with a still-unknown factor on the opposing gamete, since successful sperm entry requires compatibility between a species’ Bouncer and the sperm (42). Whether a similar mode of action (e.g., a SPACA4 interacting protein expressed in the oocyte and a possible involvement in species-specificity of sperm–egg interaction) also applies to mammals is currently unclear. Given the divergence in function of Ly6/uPAR proteins (43, 61), it is possible that SPACA4 acquired a different function from Bouncer [e.g., by acting in cis through interacting with other sperm-expressed membrane proteins or by interacting with molecules in the extracellular matrix surrounding the oocyte as suggested for its interaction with plasminogen (62)]. Moreover, one can speculate that for species with internal fertilization, a selection step determining gamete compatibility at the stage of sperm–egg interaction might be less important, since mating partner selection alone can ensure that only the selected partner’s sperm will be available for fertilization. This is not the case for species performing external fertilization who cannot guarantee by premating choice that only conspecific sperm reaches the egg (28, 63) and whose oocyte-specific expression of Bouncer could contribute to postcopulation female mate choice (also called cryptic female mate choice) (64). Thus, sperm-expressed proteins like SPACA4 could promote the efficiency of fertilization, while oocyte-expressed proteins like Bouncer could support the selection of conspecific sperm. Future experiments will be required to elucidate how SPACA4 promotes fertilization in mammals and to what extent the function and mechanism differ between SPACA4 and Bouncer.

Overall, our study on SPACA4/Bouncer highlights an interesting example of a vertebrate-specific sperm–egg interaction protein that evolved a different gene expression pattern, mode of action, and, possibly, function in fish versus mammals.

## Materials and Methods

### Mouse Lines and Husbandry.

All mouse experiments were conducted according to Austrian and European guidelines for animal research and approved by local Austrian authorities or by the Animal Care and Use Committee of the Research Institute for Microbial Diseases, Osaka University (Biken-AP-H30-01). Mice were maintained under a 10-/14-h light/dark cycle (IMP) or under a 12-h light/dark cycle (Osaka University). Wild-type mice were purchased from CLEA Japan and Japan SLC (Osaka University). The mouse strain *Tg(Acr-EGFP)1Osb* has been reported before (65).

### Generation of *Spaca4* Knockout Mice.

The mouse *Spaca4* gene consists of a single exon and maps to chromosome 7. Two strategies were used to generate *Spaca4* knockout mice: 1) CRISPR-Cas9–based gene targeting (IMP) and 2) replacement of the Spaca4-encoding exon with a neomycin selection cassette via gene targeting of embryonic stem (ES) cells (Osaka University).

#### CRISPR-Cas9–based gene targeting (resultant Spaca4 alleles: C57BL/6J-Spaca4^117del^ and C57BL/6J-Spaca4^77del^).

The *Spaca4* knockout mice were generated using CRISPR-Cas9–based gene targeting. Two guide RNAs (gRNAs) targeting the coding region of *Spaca4* were generated according to published protocols (66) by oligo annealing followed by T7 polymerase–driven in vitro transcription (gene-specific targeting oligo: SPACA4_gRNA1 and SPACA4_gRNA2; common tracer oligo: *SI Appendix*, Table S1). For gRNA injections, zygotes were isolated from superovulated donor female (C57BL/6J) mice on the day of the coagulation plug (= E0.5). To remove cumulus cells, zygotes were incubated in hyaluronidase solution (∼0.3 mg/mL). The injection mix (50 ng/µL *Cas9* mRNA [Sigma-Aldrich] and 50 ng/µL SPACA4_gRNA1 and SPACA4_gRNA2) was injected into the cytoplasm of the zygotes. Injected zygotes were incubated for at least 15 min at 37 °C and 5% CO_2_. Surviving zygotes were transferred into the oviducts of pseudopregnant recipient females. The resulting pups were genotyped using primers SPACA4_gt_F and SPACA4_gt_R (*SI Appendix*, Table S1).

#### Gene targeting in ES cells (resultant Spaca4 allele: C57BL/6N-Spaca4^tm1Osb^).

The targeting vector was constructed using pNT1.1 (https://www.ncbi.nlm.nih.gov/nuccore/JN935771). A 1.9-kb NotI-XhoI short arm fragment and a 5.1-kb PacI-MfeI long arm fragment were obtained by PCR amplification using Bacterial Artificial Chromosome (BAC) DNA (RP24-343L2) as a template. The primers used were Spaca4_targeting-s_F and Spaca4_targeting-s_R for the short arm; Spaca4_targeting-l_F and Spaca4_targeting-l_R for the long arm (*SI Appendix*, Table S1). The targeting construct was linearized with ClaI and electroporated into EGR-G101 [C57BL/6N-Tg(*CAG/Acr-EGFP*)] ES cells (67). Potentially targeted ES cell clones were separated by positive/negative selection with G418 and ganciclovir. Correct targeting of the *Spaca4* allele in ES cell clones and germ-line transmission were determined by PCR. Screening primers used were Spaca4_screening+gt#781 and Spaca4_screening+gt#5081 for the short arm; Spaca4_screening+gt#5173 and Spaca4_screening+gt#678 for the long arm (*SI Appendix*, Table S1). The mutant ES clones were injected into eight-cell stage ICR embryos, and chimeric blastocysts were transferred into the uterine horns of pseudopregnant ICR females the next day. To confirm germ-line transmission, chimeric males were mated with B6D2F1 females. Offspring from heterozygous intercrosses were genotyped by PCR. The genotyping primers used were Spaca4_gt#5269, Spaca4_gt#5298, and Spaca4_screening+gt#781 (*SI Appendix*, Table S1). Two bands, a 0.3-kb band as the wild-type allele and a 0.5-kb band as the knockout allele, were amplified by PCR.

### Genotyping: Extraction of gDNA from Mouse Ear Clips and Genotyping PCR.

Mice were genotyped at weaning age (around 3 wk) using ear clips. gDNA was extracted from the ear clips using one of the following three alternative protocols. According to one protocol, ear clips were lysed by incubation in 25 µL QuickExtract Solution (QE09050, Lucigen) at 65 °C and shaking at 600 rpm for 30 min. According to a second protocol, ear clips were lysed in 100 µL lysis buffer (0.1 M NaCl, 0.5% sodium dodecyl sulfate (SDS), 10 mM Tris pH 8.0, 0.25 mM ethylenediaminetetraacetic acid (EDTA), 2 µg/µL proteinase K) at 55 °C for 3 h. To precipitate the cellular debris, 60 µL NaCl was added and the sample was centrifuged at 21,000 × *g* for 10 min at 4 °C. The supernatant was centrifuged a second time (21,000 × *g*, 10 min at 4 °C). To precipitate the DNA, 160 µL cold 100% ethanol was added, and the sample was centrifuged again (21,000 × *g*, 10 min at 4 °C). The pellet was washed a second time using 250 µL 75% ethanol (centrifugation at 21,000 × *g*, 5 min at 4 °C). The cleaned DNA pellet was dried at 37 °C for 5 to 15 min and solubilized in 50 µL ddH_2_O. According to a third protocol, a commercial lysis buffer was used to extract the gDNA from ear clippings (DirectPCR Lysis Reagent [Mouse Tail], ViagenBiotech). A total of 200 µL commercial lysis buffer and 1 µL 100 mg/mL proteinase K were added to each ear clip and incubated at 55 °C under shaking (600 rpm) overnight. For heat inactivation, the samples were heated to 85 °C for 45 min under vigorous shaking (800 rpm).

To amplify the *Spaca4* coding region, standard Taq Polyermase or Q5 Hot Start polymerase (New England Biolabs) was used with primers SPACA4_gt_F and SPACA4_gt_R (*SI Appendix*, Table S1) according to the manufacturer’s protocol. The size of the PCR product was analyzed on a 2% agarose gel.

### Identification of the Nature of the *SPACA4*^*77del*^ and *SPACA4*^*117del*^ Mutations.

To analyze the nature of the *Spaca4* mutations, the PCR products from the genotyping of the first outcross (generation F1) were cloned into the cloning vectors provided by the StrataClone PCR cloning kit (Stratagene). The cloning was performed according to the manufacturer’s protocol. Of the resulting bacterial colonies, 96 colonies were picked and sequenced using the primer SPACA4_gt_F (*SI Appendix*, Table S1). Two mice, one with a 77-nt and the other one with a 117-nt deletion in the coding sequence of *Spaca4*, were selected and used for further in- and outcrossing. Both deletions were also confirmed by sequencing the PCR products from the genotyping of the next generation (F2).

Wild-type *Spaca4* Open Reading Frame (ORF) (the bold sequence is deleted in the mutant *Spaca4*^*77del*^; the underlined sequence is deleted in the mutant *Spaca4*^*117del*^): Atg​gtc​ctt​ggc​tgg​cca​ctg​ctt​ctg​gtg​ttg​gtt​ctt​tgc​cca​ggt​gtg​aca​ggc​atc​aag​gac​tgc​gtc​ttc​tgt​gag​ctg​act​gac​tct​gct​cgg​tgc​cct​ggc​aca​cac​atg​cgc​tgtggg​gat​gac​gaa​gat​tgc​tt**cac​agg​cca​cgg​agt​agc​cca​ggg​tgt​ggg​gcc​cat​cat​caa​caa​agg​ctg​cgt​gca​ctc​cac​cag​ctg​tgg​ccg​cg**agg​aac​cca​tca​gct​aca​tgggc​ctc​aca​tac​agt​ctc​acc​acc​acc​tgc​tgt​tct​ggc​cac​ctt​tgc​aat​aag​ggc​act​ggc​ctt​tcc​aca​ggg​gct​acc​agc​ctg​tca​ctg​ggt​ctg​cag​ctg​ctc​ctg​ggc​ctg​ttg​ctg​ctg​ctt​caa​tac​tgg​ctg​tga.

Wild-type SPACA4 protein (underlined sequence, signal peptide; the bold sequence is deleted in the mutant *Spaca4*^*77del*^; the underlined italicized sequence is deleted in the mutant *Spaca4*^*117del*^): MVLGWPLLLVLVLCPGVTGIKDCVFCELTDSARCPGTHMRCG*DDEDCFTGHGVAQGVGPIINKGCVHSTSCGREEPISYMG*LTYSLTTTCCSGHLCNKGTGLSTGATSLSLGLQLLLGLLLLLQYWL*.

Spaca4^77del^ ORF is as follows: Atg​gtc​ctt​ggc​tgg​cca​ctg​ctt​ctg​gtg​ttg​gtt​ctt​tgc​cca​ggt​gtg​aca​ggc​atc​aag​gac​tgc​gtc​ttc​tgt​gag​ctg​act​gac​tct​gct​cgg​tgc​cct​ggc​aca​cac​atg​cgc​tgt​ggg​gat​gac​gaa​gat​tgc​tta​gga​acc​cat​cag​cta​cat​ggg​cct​cac​ata​cag​tct​cac​cac​cac​ctg​ctg​ttc​tgg​cca​cct​ttg​caa​taa.

SPACA4^77del^ protein (the bold sequence does not exist in the wild-type, as it is a consequence of the out-of-frame deletion) is as follows:

MVLGWPLLLVLVLCPGVTGIKDCVFCELTDSARCPGTHMRCGDDEDCLGTHQLHGPHIQSHHHLLFWPPLQ*.

Spaca4^117del^ ORF is as follows: Atg​gtc​ctt​ggc​tgg​cca​ctg​ctt​ctg​gtg​ttg​gtt​ctt​tgc​cca​ggt​gtg​aca​ggc​atc​aag​gac​tgc​gtc​ttc​tgt​gag​ctg​act​gac​tct​gct​cgg​tgc​cct​ggc​aca​cac​atg​cgc​tgt​ggc​ctc​aca​tac​agt​ctc​acc​acc​acc​tgc​tgt​tct​ggc​cac​ctt​tgc​aat​aag​ggc​act​ggc​ctt​tcc​aca​ggg​gct​acc​agc​ctg​tca​ctg​ggt​ctg​cag​ctg​ctc​ctg​ggc​ctg​ttg​ctg​ctg​ctt​caa​tac​tgg​ctg​tga.

SPACA4^117del^ protein is as follows: MVLGWPLLLVLVLCPGVTGIKDCVFCELTDSARCPGTHMRCGLTYSLTTTCCSGHLCNKGTGLSTGATSLSLGLQLLLGLLLLLQYWL*.

### RT-PCR and RT-qPCR of *Spaca4*.

Mouse complementary DNA (cDNA) was prepared from multiple adult tissues of ICR mice and from testes of *Spaca4* knockout mice. Total RNA was reverse-transcribed into cDNA using a SuperScript III First-Strand Synthesis System for RT-PCR (Invitrogen). The amplification conditions for PCR were 2 min at 50 °C and 30 s at 95 °C, followed by 39 cycles of 95 °C for 15 s and 60 °C for 1 min (+ plate read) (RT-qPCR: Fig. 1*C*) or 1 min at 94 °C, followed by 30 cycles of 94 °C for 30 s, 65 °C for 30 s, and 72 °C for 30 s, with a final 7-min extension at 72 °C (RT-PCR: *SI Appendix*, Fig. S2*D*), using primers targeting *Spaca4* (Spaca4_qPCR_F2 and Spaca4_qPCR_R2) and *Hprt* (HPRT_qPCR_F and HPRT_qPCR_R) (Fig. 1*C*) or *Spaca4* (Spaca4_qPCR_F1 and Spaca4_qPCR_R1) and *Gapdh* (Gapdh_RT_F and Gapdh_RT_R) (*SI Appendix*, Fig. S2*D*).

### In Vivo Fertility Assays.

Fertility assays in mice were performed according to two alternative methods: In the case of CRISPR-generated C57BL/6J-*Spaca4* alleles, C57BL/6J-*Spaca4* wild-type, heterozygous, transheterozygous (*Spaca4*^*117*del/77*del*^), or homozygous (*Spaca4*^*117*del/117del^ or *Spaca4*^*77*del/77*del*^) mutant male or female mice were caged with 2- to 4-mo-old B6129F1 wild-type mice in the evening. Females were checked for plugs every morning and separated from the males as soon as a plug could be observed. The number of pups for each female was counted within a week of birth. In the case of the mutant male mice, this procedure was repeated at least once before the mutant mice were kept caged with a B6129F1 female for 3 to 10 wk after the initial plug.

In the case of C57BL/6N-*Spaca4*^tm1Osb^ mutants, sexually mature wild-type, heterozygous or homozygous mutant male mice were caged with 2-mo-old B6D2F1 for several months, and the number of pups in each cage was counted within a week of birth. Average litter sizes are presented as the number of total pups born divided by the number of litters for each genotype.

### IVF Assays.

Before IVF, female mice were superovulated by injection of CARD HyperOva (KYD-010-EX, Cosmo Bio Co) approximately 63 h before and human chorionic gonadotropin (hCG, Chorulon) 14 to 16 h before harvesting the oocytes. IVF was performed using Toyoda, Yokoyama, Hoshi (TYH) medium, CARD MEDIUM (KYD-003-EX, Cosmo Bio Co), and CARD FERTIUP Preincubation Medium (KYD-002-EX, Cosmo Bio Co) according to the manufacturer’s protocol. Sperm was prepared from the cauda epididymides and capacitated in CARD FERTIUP medium for 1 h or TYH medium for 2 h. Oocytes from superovulated female mice (C57BL/6J) were introduced into a drop of CARD MEDIUM or TYH medium. To prepare cumulus- or zona-free oocytes (Fig. 3*C*), COCs were collected in M2 medium (MR-015P-5D, Merck) and treated with 300 µg/mL hyaluronidase (H3884, Sigma-Aldrich) until the cumulus cells were removed, and cumulus cell-free oocytes were washed in M2. For ZP removal, the cumulus cell–free oocytes were moved to a droplet of acidified Tyrode’s solution (T1788, Sigma-Aldrich) for a few seconds, then washed with M2 and finally transferred into CARD MEDIUM. Afterward, the preincubated sperm was added to the differently treated oocytes for fertilization. Sperm and eggs were incubated at 37 °C and 5% CO_2_ and washed 3 h after incubation. Fertilization rates were recorded by counting the number of two-cell stage embryos on the next day.

### Sperm Number and Motility Analyses.

The number (*SI Appendix*, Fig. S4*D*) and overall motility (motile, progressive motile; *SI Appendix*, Fig. S4*E*) of sperm were measured using the computer-assisted sperm analysis system CEROS II animal (Hamilton Thorne) according to the manufacturer’s protocol. In brief, 1 µL sperm was diluted 1:200 in Dulbecco's phosphate-buffered saline (DPBS, MR-006C, Merck), and then overall motility was assessed on a CEROS II.

To quantify sperm motility under IVF conditions (*SI Appendix*, Fig. S6*C*), cauda epididymal spermatozoa were squeezed out and then dispersed in TYH (for sperm motility and IVF). After incubation of 10 and 120 min in TYH, sperm motility patterns were examined using the CEROS II sperm analysis system (58, 68).

### Assessment of Sperm Binding to the ZP.

The sperm ZP-binding assay was performed as described previously (69). In brief, cumulus cells of oocytes were removed by treatment with bovine testicular hyaluronidase (175 U/mL; Sigma-Aldrich) for 5 min. In the TYH medium, cumulus-free oocytes were mixed and incubated for 2 h with spermatozoa of the indicated genotypes and fixed with 0.25% glutaraldehyde for 30 min. The bound spermatozoa were observed with an Olympus IX73 microscope (Olympus) (Fig. 3*A*) or an IX-70 fluorescent microscope (Olympus) (Fig. 3*B* and *SI Appendix*, Fig. S6 *A* and *B*). IZUMO1 was used as a marker of the acrosome reaction in spermatozoa bound to the ZP. After fixation, the oocytes were incubated with rat anti-IZUMO1 antibody (KS64-125) for 30 min, followed by incubation with goat anti-rat immunoglobulin G (IgG) Alexa Fluor 488 for 30 min. The AR spermatozoa were observed with a BZ-X710 fluorescent microscope (Keyence) (Fig. 3*B* and *SI Appendix*, Fig. S6 *A* and *B*).

### Generation of Anti-Mouse SPACA4 Monoclonal Antibodies.

Monoclonal antibody production was performed as described previously (69). Briefly, cauda epididymal sperm were collected from the ICR mouse strain, and sperm heads and tails were separated by mild sonication on ice. The sample was layered on a discontinuous sucrose gradient. Sperm heads were in the pellet after ultracentrifugation. The pellet fraction was used as antigen. Three female Wistar rats were immunized using the sperm head solution. Spleen cells were harvested after the third immunization and fused with mouse myeloma cells (P3U1). The hybridomas were screened by an enzyme-linked immunosorbent assay, and the positive clones were grown. Their supernatants were used for immunostaining and immunoblot analyses.

### Immunostaining of Mouse Sperm.

Immunostaining of mouse sperm was performed as described previously (70). Briefly, all samples were mounted on glass slides and dried. After washing with phosphate-buffered saline (PBS), slides were blocked with 10% Normal Goat Serum/PBS (B6J) or 10% Newborn Calf Serum (NBCS)/PBS (B6D2F1) for 1 h and incubated with primary antibodies [rat monoclonal anti-mouse SPACA4 (KS139-281) (71), rat monoclonal anti-mouse IZUMO1 (KS64-125) (71, 72), and rabbit anti-mouse IZUMO1 (4)] in blocking buffer at 4 °C overnight. After washing with PBS + 0.1% Tween 20 (PBS-T) (B6J) or 10% NBCS/PBS-T (B6D2F1), the slides were incubated with secondary antibodies (goat anti-rat IgG Alexa Fluor 488 [A11006], goat anti-rat IgG Alexa Fluor 546 [A11081], and goat anti-rabbit IgG Alexa Fluor 488 [A11008], all purchased from Thermo Fisher Scientific) in blocking buffer for 1 h. After washing with PBS containing 0.05% Tween-20, the slides were observed under a fluorescence microscope (B6J: Axio Imager.Z2, Zeiss; B6D2F1: IX70, Olympus). The mouse strain *Tg(Acr-EGFP)1Osb* was used to identify spermatozoa with unreacted acrosomes (65).

### Western Blotting.

Immunoblot analysis from mouse sperm was performed as described previously (69). Briefly, sperm samples were collected from the cauda epididymis and vas deferens. These samples were homogenized in lysis buffer containing 1% Triton X-100 and 1% protease inhibitors (Nacalai Tesque), centrifuged, and the supernatants were collected. Protein lysates were resolved by SDS/polyacrylamide gel electrophoresis (PAGE) under reducing condition and transferred to polyvinylidene fluoride (PVDF) membranes (Merck Millipore). After blocking, blots were incubated with primary antibodies [rat monoclonal anti-mouse SPACA4 (KS139-281) (71), mouse monoclonal anti-ADAM3 sc-365288 (Santa Cruz Biotechnology), rat monoclonal anti-IZUMO1 KS64-125 (71, 72), goat polyclonal anti-BASIGIN/EMMPRIN sc-9757, or mouse monoclonal anti-BASIGIN/EMMPRIN sc-46700 (Santa Cruz Biotechnology)] overnight at 4 °C and then incubated with secondary antibodies conjugated with horseradish-peroxidase (HRP) (HRP-conjugated goat anti-rat IgGs [112-035-167] and HRP-conjugated goat anti-mouse IgGs [115-036-062] [Jackson ImmunoResearch Laboratories]). The detection was performed using an enhanced chemiluminescence (ECL) plus Western blotting detection kit (GE Healthcare) and Chemi-Lumi One Super (Nacalai Tesque).

### Protein Sequence Alignments of Fertility Factors.

Protein sequences of human CD9 (P21926), IZUMO1 (Q8IYV9), SPACA6 (W5XKT8), JUNO/IZUMO1R (A6ND01), TMEM95 (Q3KNT9), FIMP (Q96LL3), and mouse LLCFC/SOF1 (Q9D9P8) were downloaded from uniprot (https://www.uniprot.org/) and searched via the National Center for Biotechnology Information (NCBI) protein BLAST blastp (https://blast.ncbi.nlm.nih.gov/Blast.cgi?PAGE=Proteins) for homologous protein sequences in *Mus musculus* (taxid:10090) or *Homo sapiens* (taxid:9606), reptiles (taxid:8459), *Xenopus* (taxid:8353), fugu (taxid:31032), *Danio rerio* (taxid:7955), platypus (taxid:9258), and armadillo (taxid:9359). If no homologous protein sequence was found, the search was extended to translated nucleotide sequences using tblastn. Protein sequence alignments were generated with clustal omega (https://www.ebi.ac.uk/Tools/msa/clustalo/) using default parameters and plotted with JalView (https://www.jalview.org/).

### Bioinformatic Analyses of Ly6/uPAR Genes.

Our analysis of the mouse Ly6/uPAR family considered 66 mouse genes annotated in the Ensembl release 79 of GRCm38. The Ensembl release was chosen to match the mouse expression data (73). The set of genes was selected based on sequence similarity to the mouse Ly6/uPAR family published in ref. 43 and domain similarity searches with Ly6/uPAR domain Hidden Markov Models (HMMs) against the Ensembl proteome (42). For defining the set of human orthologs of the mouse Ly6/uPAR genes, mouse gene symbols were used to query against Diopt version 8.0 (74), and hits were only considered with Best.Score, Best.Score.Reverse, and Rank “high.” Mouse expression data were obtained from ref. 73. Human expression data were obtained from GTEx version 8 (www.gtexportal.org; GTEx_Analysis_2017-06-05_v8_RNASeQCv1.1.9_gene_median_tpm). *Xenopus laevis* expression data were derived from the NCBI Gene Expression Omnibus (GEO) entry GSE73419 (75). *Danio rerio* expression data were derived from GEO entries GSE111882 (testis, ovary, and mature oocytes) (42), GSE147112 (oogenesis and mature oocytes) (76), and GSE171906 (adult tissues) (11). Heatmaps were used to illustrate the relative expression levels of genes across tissues. To this end, published library size and length-corrected expression values (transcript per million mapped reads (TPM)/fragments per kilo base per million mapped reads (FPKM)) were obtained; after square-root–based variance stabilizing transformation, the obtained values were centered and scaled per gene (z-score).

### Statistical Analysis.

Statistical analysis was performed with the GraphPad Prism 7 software. Statistical tests are detailed in each figure legend. Differences were considered significant at *P < 0.05, **P < 0.01, and ***P < 0.001; n.s., not significant). Error bars represent SD. Figure legends indicate the number of *n* values and number of experiments for each analysis.

## Supplementary Material

Supplementary File

## Data Availability

Previously published data were used for this work that are available on the publicly accessible database GEO [*Danio rerio* expression data were derived from GEO entries GSE111882 (testis, ovary, and mature oocytes) (42), GSE147112 (oogenesis and mature oocytes) (76), and GSE171906 (adult tissues) (11)]. The *Spaca4* knockout mice, *C57BL/6N-Spaca4^tm1Osb^*, were deposited into the RIKEN BioResource Center (https://mus.brc.riken.jp/en/) and are available to the scientific community. All data are included in the article and/or *SI Appendix*, and are freely available to the scientific community.
