# Supplementary material for: The conserved fertility factor SPACA4/Bouncer has divergent modes of action in vertebrate fertilization
Source: Proc Natl Acad Sci U S A. 2021 Sep 23;118(39):e2108777118. doi: 10.1073/pnas.2108777118 (PMC8488580; doi:10.1073/pnas.2108777118)
Supplement: Supplementary File [file pnas.2108777118.sapp.pdf]

PNAS
www.pnas.org

Supplementary Information for

The conserved fertility factor SPACA4/Bouncer has divergent modes of action in vertebrate fertilization

Yoshitaka Fujihara[#], Sarah Herberg[#], Andreas Blaha, Karin Panser, Kiyonori Kobayashi, Tamara Larasati, Maria Novatchkova, Hans-Christian Theußl, Olga Olszanska, Masahito Ikawa* & Andrea Pauli*

Corresponding authors: Masahito Ikawa & Andrea Pauli

Email: ikawa@biken.osaka-u.ac.jp, andrea.pauli@imp.ac.at

This PDF file includes:

Figures S1 to S6
Table S1
SI References

Figure S1

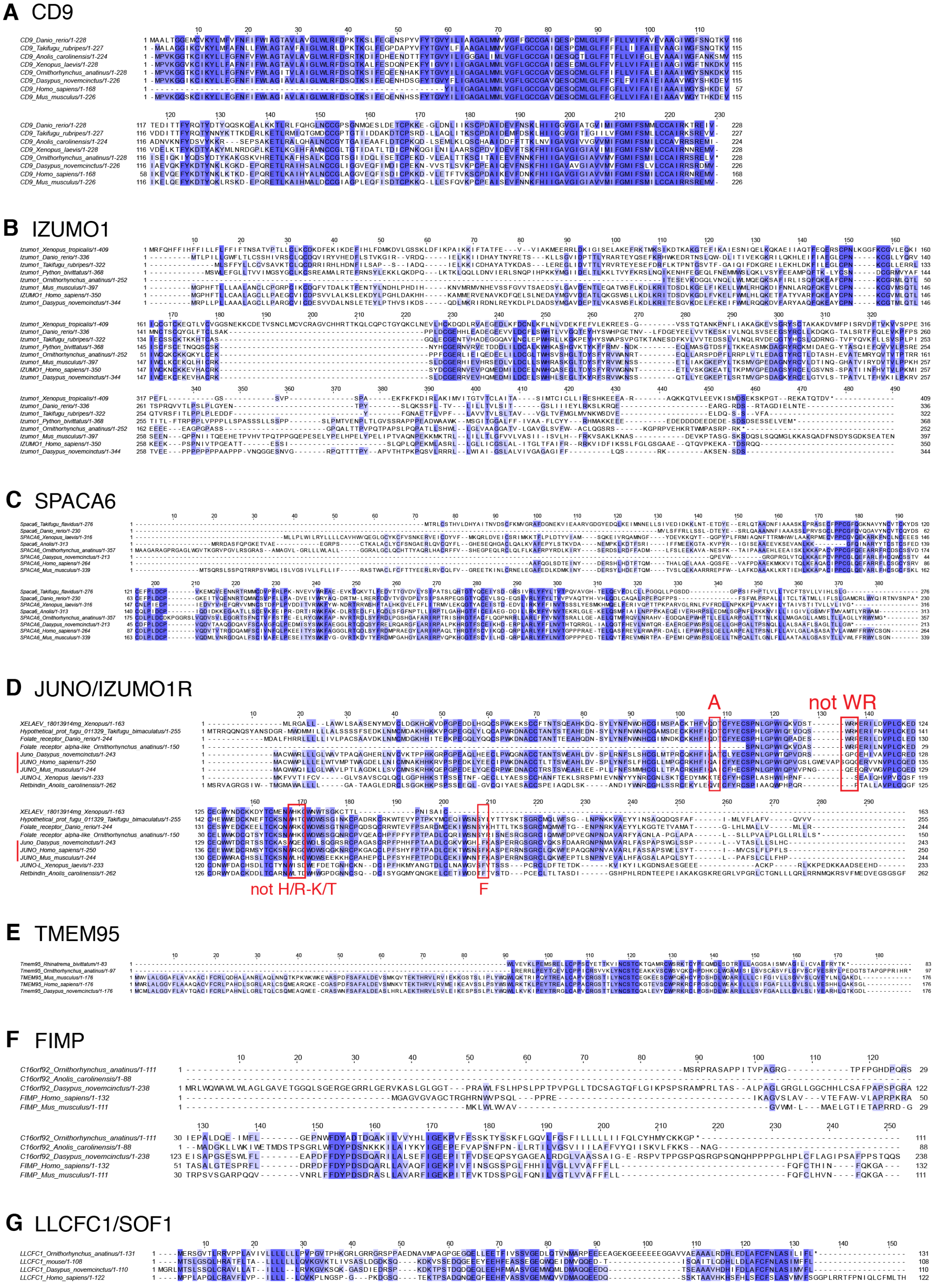
A CD9
B IZUMO1
C SPACA6
D JUNO/IZUMO1R
A
not WR
not H/R-K/T
F
E TMEM95
F FIMP
G LLCFC1/SOF1

Fig. S1. Analysis of the conservation of mammalian fertility factors across vertebrates. (*A*-*G*) Protein sequence alignments of mammalian fertility factors known to be essential for sperm-egg interaction in mice. (*A*) CD9, (*B*) IZUMO1, (*C*) SPACA6, (*D*) JUNO/IZUMO1R, (*E*) TMEM95, (*F*) FIMP, (*G*) LLCFC1/SOF1. To *D*: Although folate receptor homologs (JUNO/IZUMO1R belong to the folate receptor family) are found outside of mammals, amino acids defining the JUNO/IZUMO1R (highlighted in red) are different outside of mammals [1]. Based on this definition, JUNO/IZUMO1R homologs are only present in mammals (red line).

Figure S2

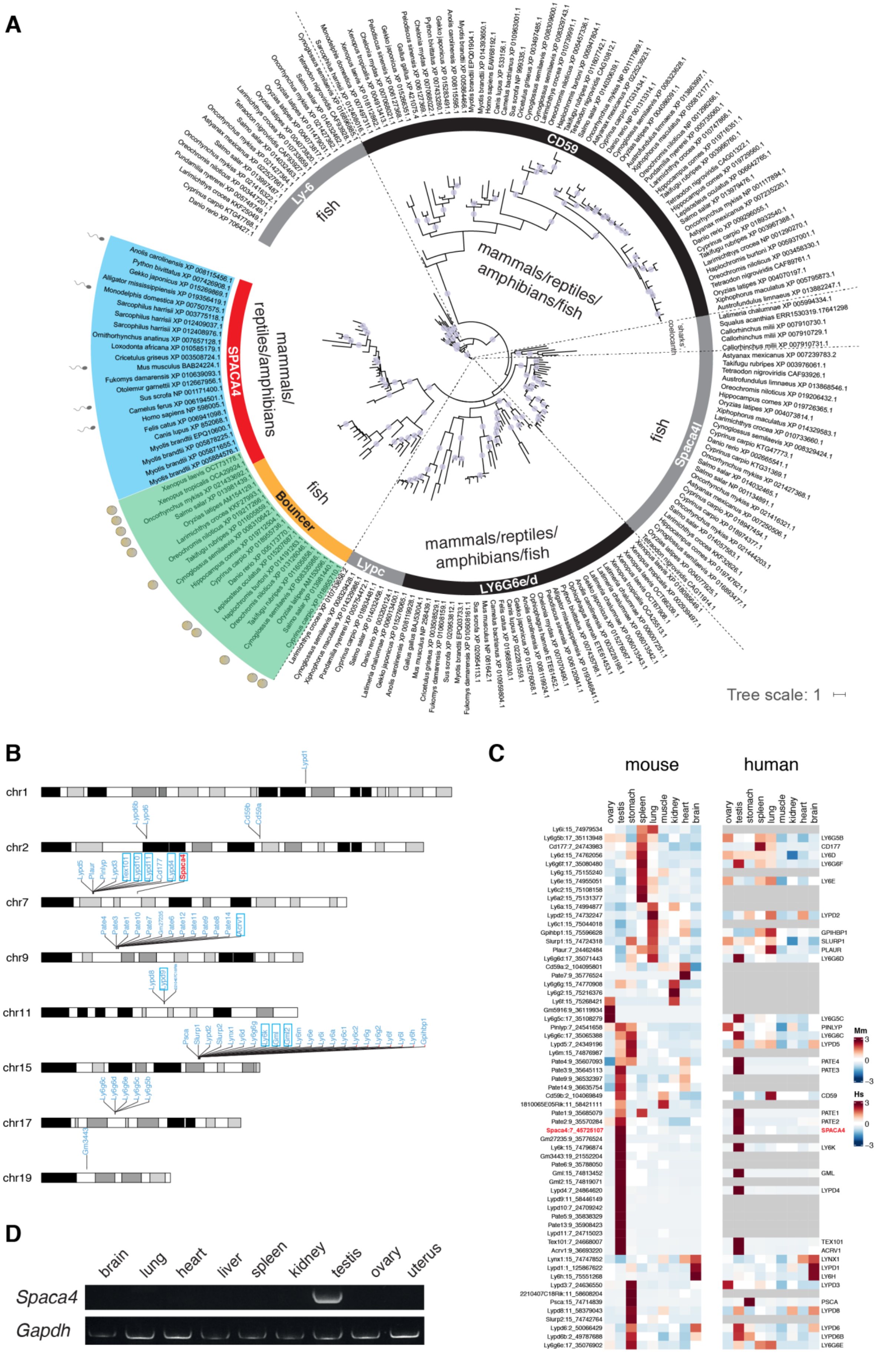
A
CD59
Ly-6
fish
mammals/reptiles/amphibians/fish
sharks
coelacanth
SPACA4
mammals/reptiles/amphibians
Bouncer
Lypc
LY6G6e/d
mammals/reptiles/amphibians/fish
Spaca4l
Tree scale: 1
B
chr1
chr2
chr7
chr9
chr11
chr15
chr17
chr19
Spaca4
C
mouse
human
ovary
testis
stomach
spleen
lung
muscle
kidney
heart
brain
Mm
Hs
3
0
−3
Spaca4:7_45725107
SPACA4
D
brain
lung
heart
liver
spleen
kidney
testis
ovary
uterus
Spaca4
Gapdh

Fig. S2. Expression analysis of mammalian Ly6/uPAR proteins. (*A*) Phylogenetic tree of selected groups of Ly6/uPAR proteins across vertebrates. Maximum-likelihood phylogenetic tree based on Ly6/uPAR protein sequence alignments across vertebrates (adapted from [2]). Branches supported by ultrafast bootstrap values (>=95%) are marked with a blue dot. SPACA4 (red) is present in mammals, reptiles and amphibians; Bouncer (yellow) is present in fish. The sperm and egg symbols next to selected species' genes indicate expression in testis (blue) or ovaries (green). (*B*) Genomic localization of Ly6/uPAR genes in mice. Ly6/uPAR genes are clustered in the genome. Only chromosomes containing at least one Ly6/uPAR gene are shown. Testis-expressed genes are highlighted with a blue box; *Spaca4* is highlighted in red. (*C*) Mouse and human Ly6/uPAR proteins are expressed in various tissues, including in gametes (extension of **Fig. 1B** including human orthologs). Heatmaps of expression levels of homologous mouse (Mm; left) and human (Hs; right) Ly6/uPAR proteins (homologous proteins are shown in one row) across various adult tissues. Heatmaps are color-coded based on z-scores of the normalized gene expression values (average of the square-root) of RNA-Seq data from murine and human adult tissues [3] (www.gtexportal.org). Adult tissues are indicated at the bottom. Gene names are given on the left (mouse) and right (human) of the corresponding heat-maps. Numbers behind gene names indicate chromosome locations in mice or humans. *Spaca4* is highlighted in red. (*D*) Murine *Spaca4* is expressed in the male but not the female germline. RT-PCR for *Spaca4* from cDNA of the indicated mouse tissues relative to the housekeeping gene *Gapdh*. The template cDNA was generated from a mixture of the tissue from 3-4 different mice.

Figure S3

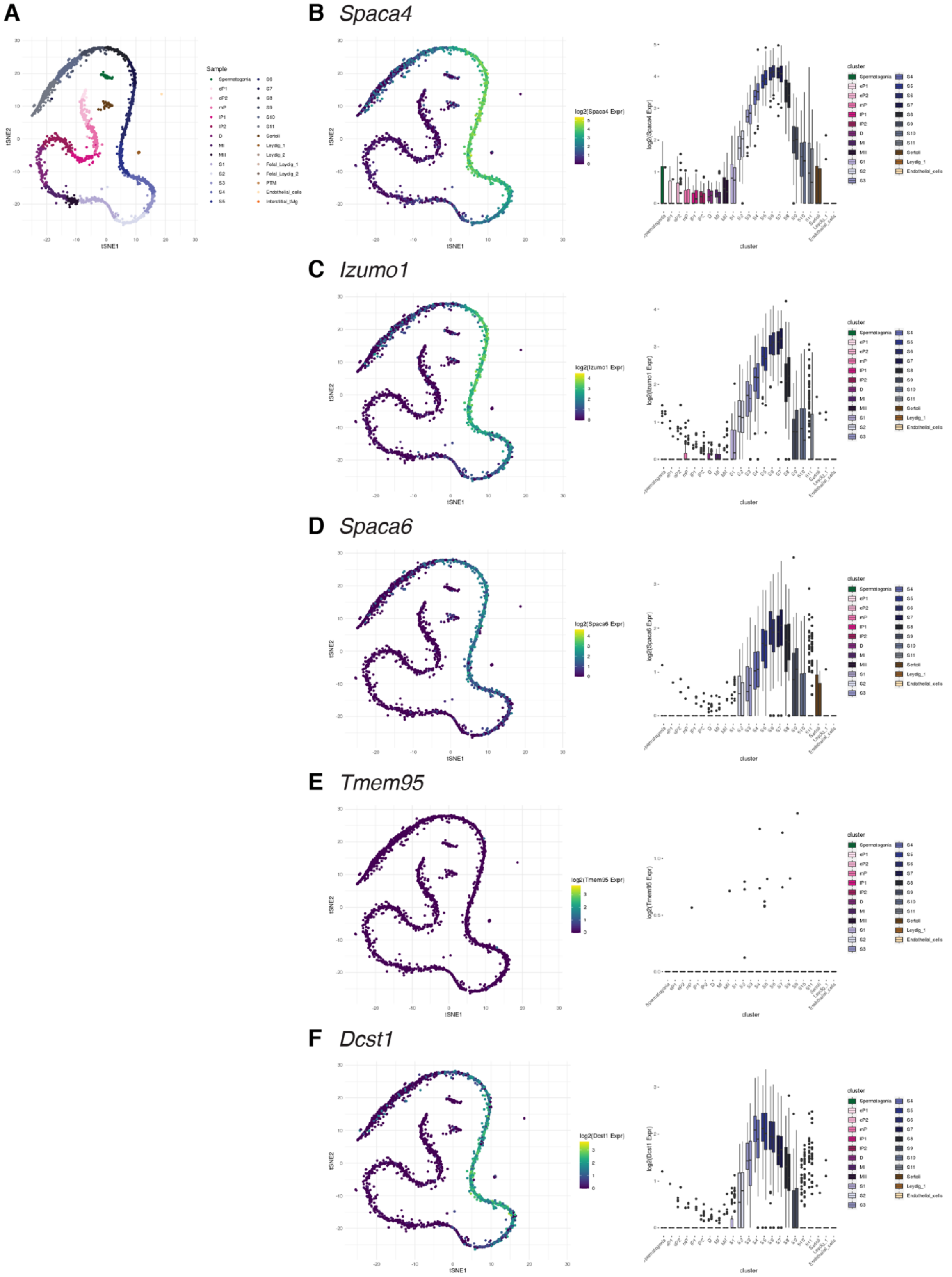

Fig. S3. Analysis of the expression of selected mammalian fertility factors during murine spermatogenesis. (*A*) Overview of the different stages of spermiogenesis detected via scRNA-Seq

(single-cell RNA-Seq), represented in a tSNE1-tSNE2 (t-distributed stochastic neighbor embedding) plot. (*B-F*) Expression patterns and values of selected fertility factors during murine spermatogenesis were derived from published single-cell RNA-Seq data [44]. The web-browser version https://marionilab.cruk.cam.ac.uk/SpermatoShiny/ was used to generate the plots for (*B*) *Spaca4*, (*C*) *Izumo1*, (*D*) *Spaca6*, (*E*) *Tmem95* and (*F*) *Dcst1*. The *Spaca4* temporal expression pattern resembles *Izumo1's, Spaca6's,* and *Dcst1's* pattern.

Figure S4

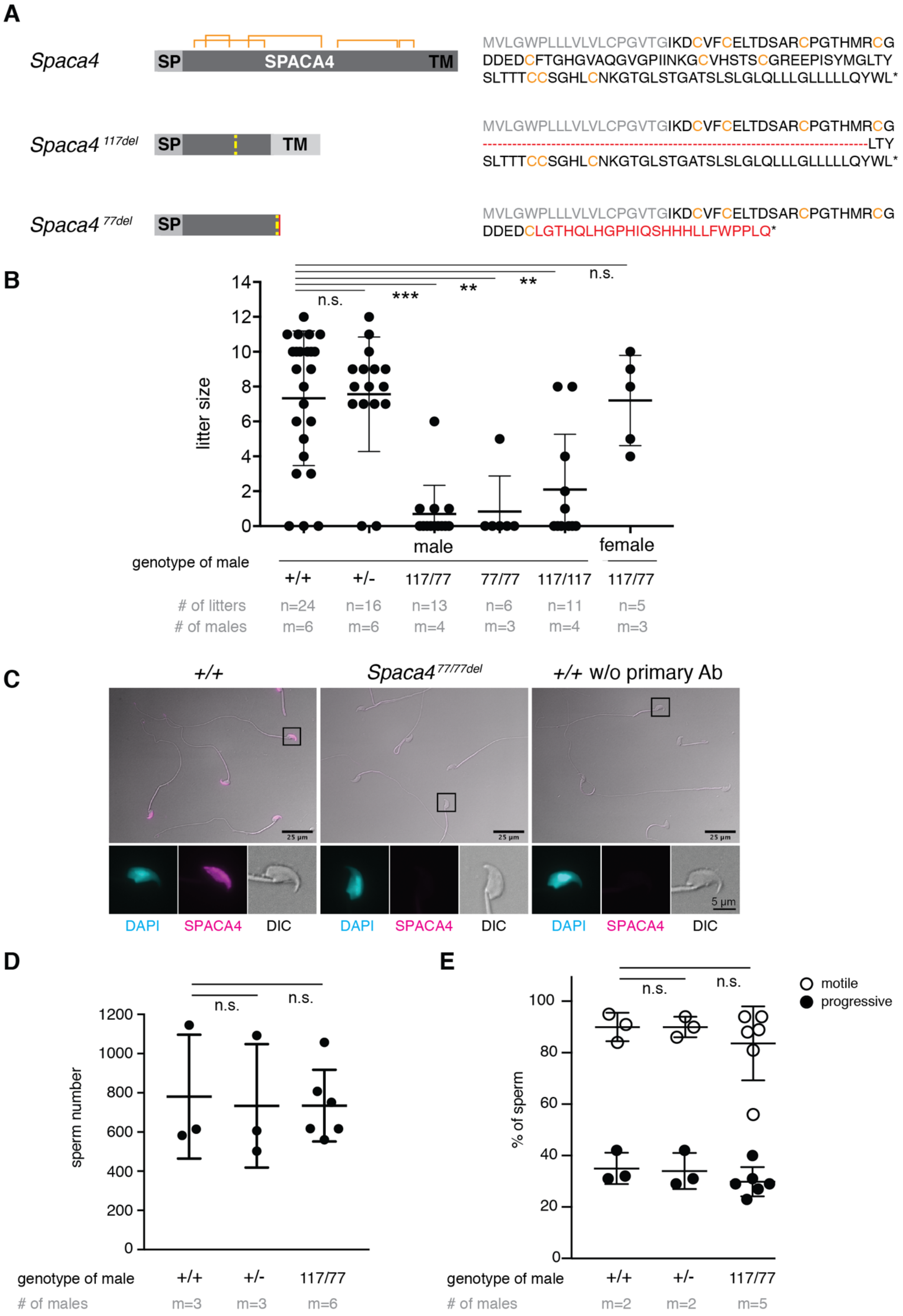

A
Spaca4
SP
SPACA4
TM
MVLGWPLLLVLVLCPGVTGIKDCVFCELTDSARCPGTHMRCG
DDEDCFTGHGVAQGVGPIINKGCVHSTSCGREEPISYMGLTY
SLTTTCCSGHLCNKGTGLSTGATSLSLGLQLLLGLLLLLQYWL*
Spaca4 117del
SP
TM
MVLGWPLLLVLVLCPGVTGIKDCVFCELTDSARCPGTHMRCG
--LTY
SLTTTCCSGHLCNKGTGLSTGATSLSLGLQLLLGLLLLLQYWL*
Spaca4 77del
SP
MVLGWPLLLVLVLCPGVTGIKDCVFCELTDSARCPGTHMRCG
DDEDCLGTHQLHGPHIQSHHHLLFWPPLQ*
B
litter size
0
2
4
6
8
10
12
14
n.s.
***
**
**
n.s.
male
female
genotype of male
+/+
+/-
117/77
77/77
117/117
117/77
# of litters
n=24
n=16
n=13
n=6
n=11
n=5
# of males
m=6
m=6
m=4
m=3
m=4
m=3
C
+/+
Spaca4 77/77del
+/+ w/o primary Ab
25 µm
5 µm
DAPI
SPACA4
DIC
D
sperm number
0
200
400
600
800
1000
1200
n.s.
n.s.
genotype of male
+/+
+/-
117/77
# of males
m=3
m=3
m=6
E
% of sperm
0
20
40
60
80
100
n.s.
n.s.
motile
progressive
genotype of male
+/+
+/-
117/77
# of males
m=2
m=2
m=5

Fig. S4. Generation and phenotypic analysis of *Spaca4* knockout mice. (*A*) Overview of the C57BL/6J-*Spaca4* knockout alleles generated by CRISPR/Cas9-mediated targeted mutagenesis. (Left) Schematics of the wild-type and knockout alleles. Yellow dashed lines indicate the site of the deletions. Predicted disulfide bridges are indicated in orange. SP, signal peptide; TM, transmembrane region. (Right) Amino acid sequences of the protein products that are predicted to be produced from the different alleles. One mutant allele contains a 117-nt in-frame deletion after amino acid 42. The other allele contains a 77-nt out-of-frame deletion after amino acid 47. Cysteines predicted to form disulfide bridges are indicated in orange; the SP sequence is shown in grey; the out-of-frame additional protein sequence in the 77del allele is shown in red. (*B*) *Spaca4* knockout male mice are sub-fertile. Litter sizes of C57BL/6J-*Spaca4* wild-type (+/+), heterozygous (+/-), transheterozygous (117/77) and homozygous (77/77 or 117/117) males of the indicated allele combinations caged with B6129F1 wild-type females, or B6129F1 wild-type males caged with transheterozygous (-/-) females. Successful mating was confirmed by plug checks. Data are means ± SD. ***$p < 0.0001$, **$p < 0.001$ (Kruskal-Wallis test with Dunn multiple-comparisons test); n.s., not significant. n = number of litters; m = number of male mice tested. (*C*) SPACA4 protein is absent in morphologically normal sperm of *Spaca4* knockout mice. Immunostaining of sperm detects SPACA4 protein (magenta) under permeabilizing conditions in the head of sperm derived from wild-type (+/+) but not *Spaca4* knockout (77/77del) mice. DAPI (cyan) staining labels the sperm nucleus. A control immunostaining in which the primary antibody was omitted is shown on the right. DIC, differential interference contrast image. Scale bar, 25 µm. (*D*) Sperm number is normal in *Spaca4* knockout males. Sperm number of *Spaca4* wild-type (+/+), heterozygous (+/-) and transheterozygous (-/-) males. Data are means ± SD. n.s., not significant (one-way ANOVA with Dunnett's multiple-comparisons test). m = number of male mice tested. (*E*) Overall sperm motility is not affected in *Spaca4* knockout males. Sperm motility of *Spaca4* wild-type (+/+), heterozygous (+/-) and transheterozygous (-/-) males. Shown is the percentage of sperm that was motile (open circles, comparison +/+ versus -/-: $p = 0.65$) and progressively motile (closed circles, comparison +/+ versus -/-: $p = 0.41$). Data are means ± SD. n.s., not significant (One-way ANOVA with Dunnett's multiple-comparisons test). m = number of male mice tested.

Figure S5

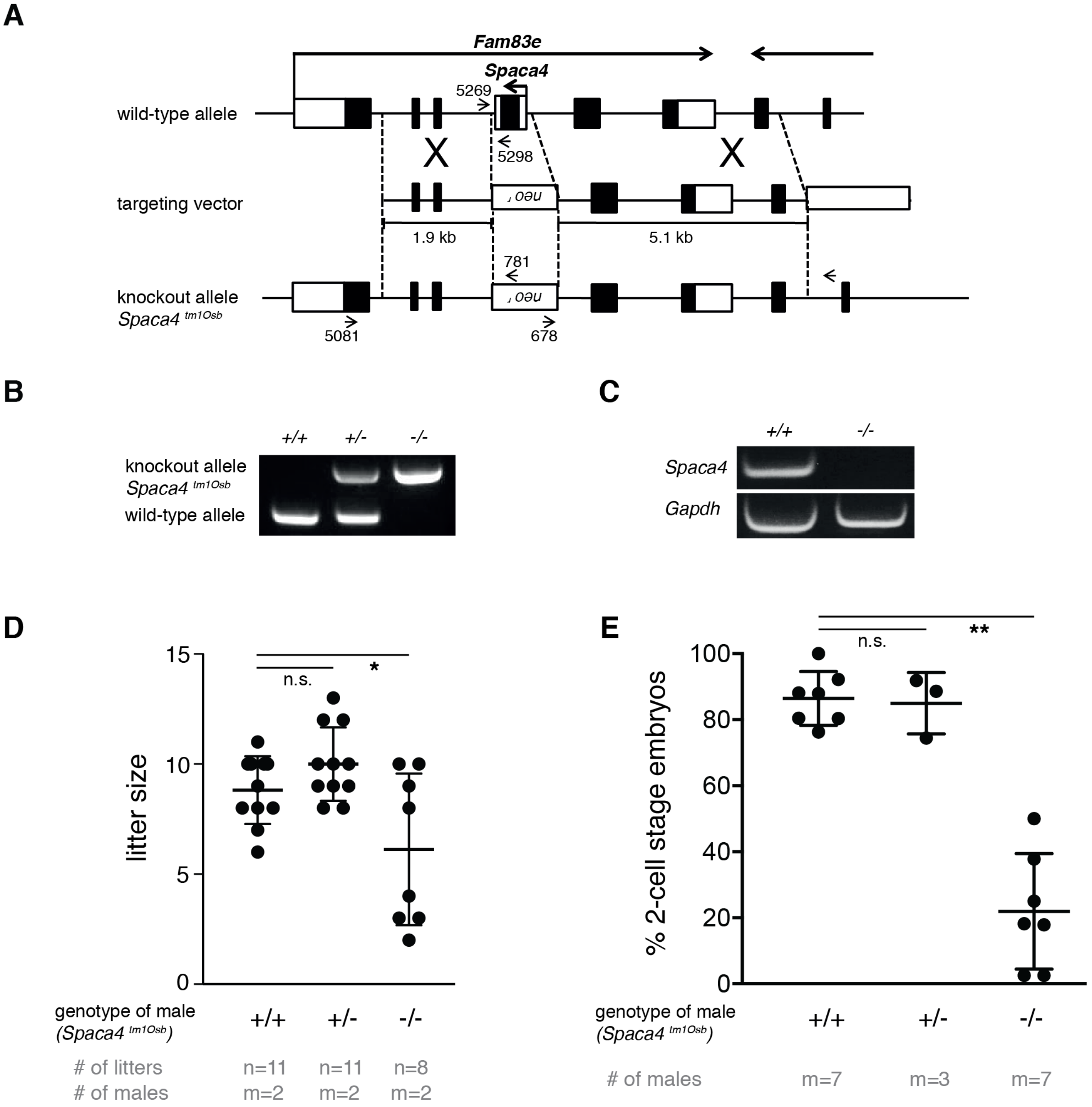

Fig. S5. Male *Spaca4* knockout mice are sub-fertile. (*A*) Targeted disruption of the murine *Spaca4* gene to generate *Spaca4*$^{\mathrm{tm1Osb}}$. To disrupt the *Spaca4* gene, the single exon was replaced with a neomycin resistance cassette (*neo*), and a thymidine kinase cassette (*tk*) was used for negative selection. Labeled arrows indicate primer binding sites. (*B*) Genotyping of *Spaca4*$^{\mathrm{tm1Osb}}$ knockout mice. Both the wild-type allele (a 0.3-kb band) and the knockout allele (a 0.5-kb band) were amplified by PCR, using primers #5269 and #5298 for the wild-type allele and primers #781 and #5269 for the knockout allele. (*C*) RT-PCR analysis of testis in wild-type and *Spaca4*$^{\mathrm{tm1Osb}}$ knockout mice. The *Spaca4*-specific 236-nt band was amplified from wild-type but not from

Spaca4^{tm1Osb} knockout testis. *Gapdh* was used as a control. **(***D-E*) *Spaca4*^{tm1Osb} knockout mice are sub-fertile. (*D*) *Spaca4*^{tm1Osb} knockout males (-/-) copulated normally but were sub-fertile compared to wild-type (+/+) and heterozygous *Spaca4*^{tm1Osb} (+/-) mutant males (comparison +/+ versus -/-: p = 0.0333, unpaired t-test). Data are means ± SD. n.s., not significant. n = number of litters; m = number of male mice tested. (*E*) *In vitro* fertilization of oocytes from wild-type females using sperm from wild-type (+/+), heterozygous *Spaca4*^{tm1Osb} (+/-) or homozygous *Spaca4*^{tm1Osb} (-/-) males. Plotted is the percentage of 2-cell stage embryos as a measure of successful fertilization. Data are means ± SD. p = 0.003 (Kruskal-Wallis test with Dunn multiple-comparisons test); n.s., not significant. m = number of males tested.

Figure S6

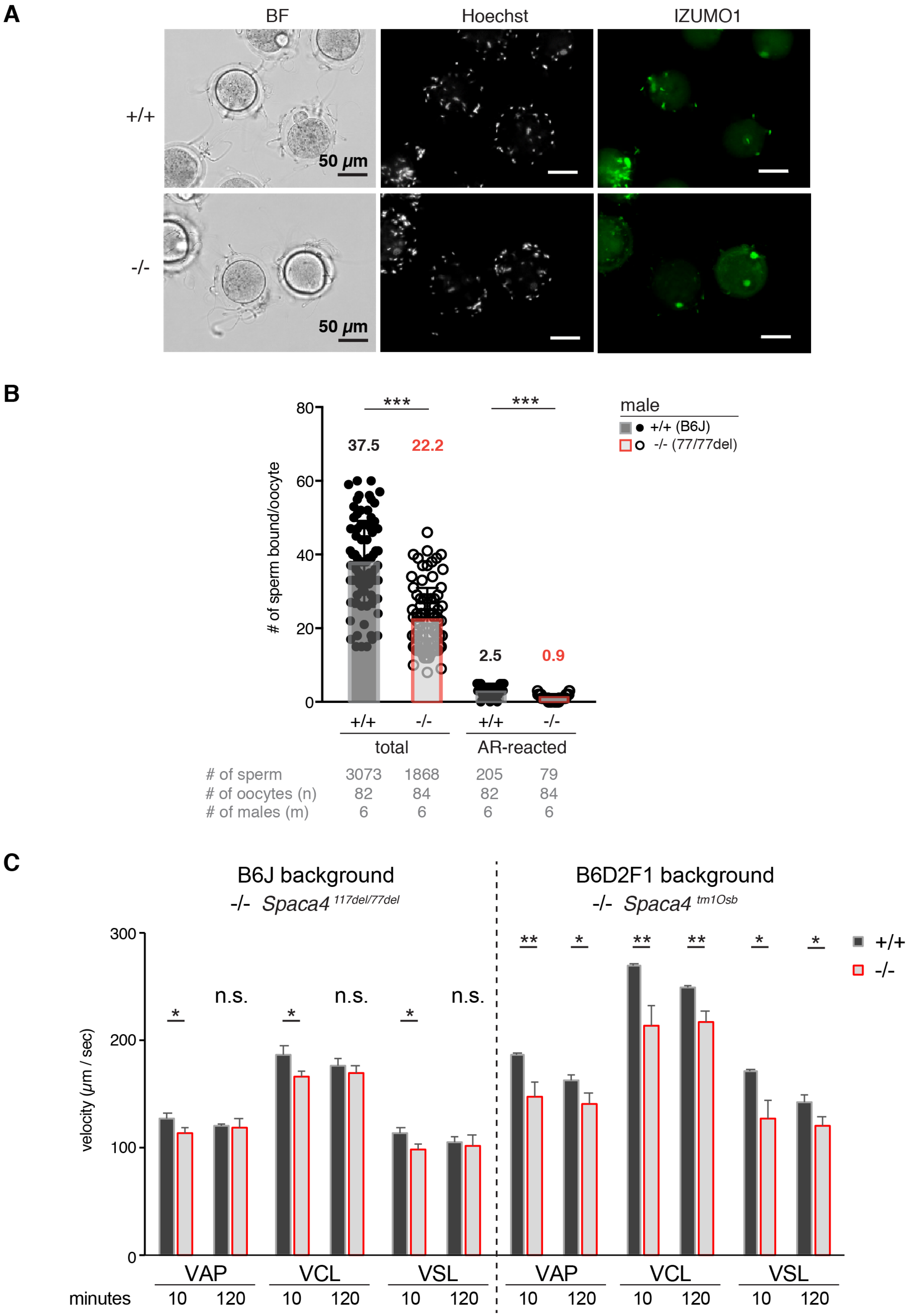

A
BF
Hoechst
IZUMO1
+/+
-/-
50 μm
50 μm
B
male
+/+ (B6J)
-/- (77/77del)
# of sperm bound/oocyte
***
***
37.5
22.2
2.5
0.9
total
AR-reacted
# of sperm 3073 1868 205 79
# of oocytes (n) 82 84 82 84
# of males (m) 6 6 6 6
C
B6J background
-/- Spaca4 117del/77del
B6D2F1 background
-/- Spaca4 tm1Osb
velocity (μm / sec)
n.s.
VAP
VCL
VSL
minutes
10
120
+/+
-/-

Fig. S6. *Spaca4* knockout sperm has reduced binding capacity to ZP-containing wild-type oocytes. (*A*) Assessment of the number of total sperm bound, acrosome-intact sperm bound (IZUMO1-staining negative) and acrosome-reacted sperm bound (IZUMO1-staining positive) by immunostaining of sperm incubated with ZP-containing wild-type oocytes under non-permeabilizing conditions. Sperm was stained with anti-IZUMO1 antibodies (green) as a read-out for a successful acrosome reaction, and Hoechst (DNA label; white) to detect all bound sperm derived from wild-type (+/+) and *Spaca4* knockout (*Spaca4*$^{77/77del}$) males. BF, bright field. Scale bar, 50 µm. (*B*) Quantification of the number of sperm bound to ZP-containing wild-type oocytes. Sperm was derived from wild-type (+/+) and *Spaca4*$^{77/77del}$ (-/-) males. Total sperm bound (left); acrosome-reacted sperm bound (right). Data are means ± SD (the value of the mean is indicated). P-value is of Student's t test. Numbers of total sperm bound, oocytes (n) and males (m) tested are indicated. Related to **Fig. 3B**. (*C*) Sperm motility was assessed under IVF conditions using the computer-assisted sperm analysis system CEROS II. Sperm motility of transheterozygous B6J (117del/77del; -/-) males was compared to wild-type B6J (+/+) males (left); similarly, sperm motility of B6D2F1 *Spaca4*^{tm1Osb} knockout (-/-) males was compared to wild-type B6D2F1 (+/+) males (right) to control for possible differences in the genetic backgrounds of both mutants. Motility of spermatozoa was assessed after 10 min incubation and after 120 min incubation. Plotted is the velocity in µm/sec. VAP: average path velocity, VSL: straight line velocity, VCL: curvilinear velocity. Data are means ± SD. n.s., not significant (Student's t test). m = 3 male mice tested for each genotype.

Table S1. List of primers

name of oligo	sequence	comments
Spaca4_qPCR_F1	AGGACTGCGTCTTCTGTGAGC	qRT-PCR #1 primer for *spaca4*
Spaca4_qPCR_R1	GCCCTTATTGCAAAGGTGGCCA	
SPACA4_qPCR_F2	TGGTGTTGGTTCTTTGCCCA	qRT-PCR #2 primer for *spaca4*
SPACA4_qPCR_R2	GTAGCTGATGGGTTCCTCGC	
Gapdh_RT_F	AGTGGAGATTGTTGCCATCAACGAC	RT-PCR primer for *gapdh*
Gapdh_RT_R	GGGAGTTGCTGTTGAAGTCGCAGGA	
HPRT_qPCR_F	GAACCAGGTTATGACCTAGATTTGTT	qRT-PCR primer for HRPT
HPRT_qPCR_R	CAAGTCTTTCAGTCCTGTCCATAAT	
SPACA4_gRNA1	taatacgactcactataGGTGACGAAGATTGCTTCACgttttagagctagaaatagcaag	T7 promoter sequence - gRNA TARGETING SEQUENCE – tracerOligo annealing sequence
SPACA4_gRNA2	taatacgactcactataGGTAGCTGATGGGTTCCTCGgttttagagctagaaatagcaag	T7 promoter sequence - gRNA TARGETING SEQUENCE – tracerOligo annealing sequence
tracer_oligo	AAAAGCACCGACTCGGTGCCACTTTTTCAAGTTGATAACGGACTAGCCTTATTTTAACTTGCTATTTCTAGCTCTAAAAC	Common oligo used to generate guideRNAs by annealing and in vitro transcription (Gagnon et al., 2014)
SPACA4_gt_F	CACTACCAGCAGAACACACCT	SPACA4 genotyping
SPACA4_gt_R	AGCTCACTGTCTCTGACCGC	
Spaca4_targeting-s_F	CTCGAGACGCACATCTTTCCACATTGACG	Spaca4 short arm targeting vector
Spaca4_targeting-s_R	GCGGCCGCTAGATGCAGCTGAAGCTCACT	
Spaca4_targeting-l_F	CAATTGATCCTTCCGCCATGTGGTTTC	Spaca4 long arm targeting vector
Spaca4_targeting-l_R	TTAATTAAGCTCTTCAGTCCTCGGGTTG	
Spaca4_screening+gt#781	GCTTGCCGAATATCATGGTGGAAAATGGCC	Screening PCR for ES cell targeting (short arm); #781 is also used for genotyping the neomycin-containing *Spaca4* knockout mouse
Spaca4_screening#5081	AGGCTGTACACGTGCTCCTCTGGT	
Spaca4_screening#5173	TGGTGCTTGCCTCTCAAACCAGCTAGACTC	Screening PCR for ES cell targeting (long arm)
Spaca4_screening#678	TCTGTTGTGCCCAGTCATAGCCGAATAGCC	
Spaca4_gt#5269	GCATGTCACGAACTCTCAGGTGACACCAA	Genotyping PCR for neomycin-containing *Spaca4* knockout mouse (Osaka, Japan)
Spaca4_gt#5298	CTGCTTCAATACTGGCTGTGACCCACAAG	

SI References

1. Grayson P. 2015 Izumo1 and Juno : the evolutionary origins and coevolution of essential sperm – egg binding partners. *R. Soc. Open Sci.* **2**, 1–11. (doi:http://dx.doi.org/10.1098/rsos.150296)
2. Herberg S, Gert KR, Schleiffer A, Pauli A. 2018 The Ly6/uPAR protein Bouncer is necessary and sufficient for species-specific fertilization. *Science (80-.).* **361**, 1029–1033. (doi:10.1126/science.aat7113)
3. Li B, Qing T, Zhu J, Wen Z, Yu Y, Fukumura R, Zheng Y, Gondo Y, Shi L. 2017 A Comprehensive Mouse Transcriptomic BodyMap across 17 Tissues by RNA-seq. *Sci. Rep.* **7**, 1–10. (doi:10.1038/s41598-017-04520-z)
4. Ernst C, Eling N, Martinez-Jimenez CP, Marioni JC, Odom DT. 2019 Staged developmental mapping and X chromosome transcriptional dynamics during mouse spermatogenesis. *Nat. Commun.* **10**. (doi:10.1038/s41467-019-09182-1)
